# Anatomopathological Characterization of the Main Ocular Lesions in Green Turtles ( *Chelonia mydas* ) Along the Northern Coast of Bahia, Brazil

**DOI:** 10.1111/vop.70106

**Published:** 2025-11-10

**Authors:** Danielle Nascimento Silva, José Luís Catão‐Dias, Wendell Marcelo de Souza Perinotto, Matheus Vilardo Loés Moreira, Nayone Lantyer‐Araujo, Pedro Enrique Navas Suárez, Gustavo Rodamilans Macedo, Thaís Pires, Arianne Pontes Oriá, Alessandra Estrela‐Lima

**Affiliations:** ^1^ School of Veterinary Medicine and Zootechny Federal University of Bahia Salvador Bahia Brazil; ^2^ Laboratory of Wildlife Comparative Pathology, School of Veterinary Medicine and Animal Sciences University of São Paulo São Paulo São Paulo Brazil; ^3^ Center of Agrarian, Environmental and Biological Sciences Federal University of Recôncavo da Bahia Cruz das Almas Bahia Brazil; ^4^ MVL Patologia Veterinária Belo Horizonte Minas Gerais Brazil; ^5^ Department of Surgical and Radiological Sciences, School of Veterinary Medicine University of California Davis California USA; ^6^ Centro Universitário – FAM São Paulo Brazil; ^7^ Laboratory of Immunology and Molecular Biology Institute of Health Sciences, Federal University of Bahia Salvador Bahia Brazil; ^8^ Fundação Projeto Tamar Mata de São João Bahia Brazil

**Keywords:** eye diseases, fibropapillomatosis, sea turtles, Spirorchiidae

## Abstract

**Objective:**

This study aimed to identify and report ophthalmic and adnexal diseases found in specimens of the Green sea turtle ( *Chelonia mydas* ).

**Animal Studied and Procedures:**

Thirty‐nine animals stranded on the beaches of the north coast of Bahia, Brazil were submitted to necropsy. A total of 158 samples of the visual system (eyelids, eyes, and salt glands) from females (71.8%; 28/39) and males (28.2%; 11/39) were analyzed.

**Results:**

Samples without macro and microscopic changes counted as 30.4% (48/158) of the evaluated samples. Approximately 69.6% (110/158) had ophthalmic lesions; 92 were bilateral (24 eyelids, 44 eyes, and 24 salt glands), and 18 were unilateral. The anatomopathological evaluation of the specimens revealed predominantly neoplastic and inflammatory lesions, with fibropapillomatosis (FP) being the most frequent finding (58.9%; 93/158), followed by spirorchidiasis (46.8%; 74/158). Other ophthalmic lesions included mucopurulent conjunctivitis, ulcerative keratitis, corneal perforation, panophthalmitis, phthisis bulbi, bilateral scar tissue in the eyelids, and lithiasis in the salt glands.

**Conclusion:**

This study highlights the significance of understanding eye diseases as they can directly impact the management and preservation of sea turtles. This is particularly true for species in which the visual system plays a crucial role in feeding and migration. Therefore, this data can assist in promoting and implementing preventive and therapeutic measures for the conservation of these animals.

## 1 Introduction

Maintaining good vision is paramount for sea turtles as they rely on it for migration and food intake. Most of the ophthalmic abnormalities described in sea turtles are located in the primary and accessory lacrimal glands, eyelids, and eyes, which are fundamental structures for the nutrition and normal behavior of these animals [1, 2, 3, 4, 5].

An understanding of the visual system pathology is essential for proper diagnosis and treatment as well as the rapid establishment of health in these animals [6, 7]. Changes in the eyes and adnexa can be caused by developmental disorders, infectious agents (viral, bacterial, fungal, and parasitological), neoplastic processes, nutritional imbalances, traumatic injuries, or even injuries that accompany systemic diseases [2, 3, 4, 5].

Few reports of eye disorders or injuries in sea turtles describe catarrhal and purulent conjunctivitis, keratitis, blepharitis, corneal perforation, and chemosis in Loggerhead sea turtle ( *Caretta caretta* ) [5]; eyelid necrosis, ulcerative keratitis, anterior uveitis, and hyphema in Green turtle ( *Chelonia mydas* ), Kemp's ridley sea turtle ( *Lepidochelys kempii* ), and *Caretta caretta* caused by low environmental temperatures [8].

The literature of free‐ranging sea turtles also describes a few reports of salt gland diseases, characterizing stone formation in severely dehydrated turtles [9]. Granulomatous adenitis by spirorchid fluke, of varying severity, is also common in the stroma surrounding the central canals of the lobules [9] and heterophilic adenitis associated with systemic lesions, or described as the sole lesion responsible for stranding sea turtles, is associated with infectious agents such as *Vibrio* spp., *Citrobacter* spp., *Pseudomonas* spp., *Aeromonas hydrophila* , *Staphylococcus* sp., and *Aerococcus viridans* [1, 3, 10, 11, 12, 13, 14].

In this context, the objective of this study was to identify and report ocular abnormalities observed in 39 turtles of the *C. mydas* species stranded on the beaches of the north coast of Bahia, Brazil, submitted to anatomopathological examination.

## 2 Materials and Methods

### 2.1 Ethical Considerations

The research protocols were approved by the Animal Welfare and Ethics Committee of the School of Veterinary Medicine and Animal Science of the Federal University of Bahia (protocol n°90/2018), in accordance with the Ministry's Biodiversity Information Authorization System of the Environment of Brazil—SISBIO (processes n°64518–1 and n°64518–3) and certified by the National System for the Management of Genetic Heritage and Associated Traditional Knowledge—SISGEN (registration n° A0F349F). In addition, the study was conducted according to the bioethics guidelines stated by the Association for Research in Vision and Ophthalmology (ARVO).

### 2.2 Animals and Samples

From February 2018 to September 2019, the eyes and adnexa (upper and lower eyelids and salt gland) of 39 specimens of *C. mydas* (38 juvenile animals and one adult animal) were collected. These were free‐living animals that washed ashore on the beaches of the northern coast of Bahia state, in the area monitored by the Fundação Projeto Tamar (Praia do Forte, latitude: −12.574743 and longitude: −38.0044715), found dead, or that died during treatment and rehabilitation at the Fundação Projeto Tamar.

### 2.3 Histopathological Processing and Analysis

After cleavage of the structures (the eyes were sectioned dorsoventrally, including the optic nerve, and the eyelids and salt glands were sectioned transversely), the fragments were placed in histological cassettes in a container with 10% phosphate‐buffered formalin solution for not less than 24 h. Tissues, after adequate fixation, were placed in cassettes for processing using a routine histological paraffin‐embedding technique. Hematoxylin and eosin (HE) staining was performed on 4‐μm sections [15].

Injuries were classified into six main categories: normal or without injury; fibropapillomatosis; spirorchidiasis; phthisis bulbi; perforated eye; and lithiasis. The animals were grouped into several categories based on the observed lesions.

The histopathological analysis was performed using a Leica optical microscope with an image capture system, using 4×, 10×, 20×, and 40× objectives. All captured photomicrographs were standardized in *ImageJ* software.

## 3 Results

Thirty‐nine specimens of *C. mydas* were evaluated; 71.8% (28/39) were female, and 28.2% (11/39) were male and a total of 158 samples from the eye and adnexa were analyzed for the present study, comprising 32 eyelid, 62 globes, and 64 Harderian gland samples.

Samples with no signs of abnormalities totaled 30.4% (48/158), comprising 30 bilateral (4 eyelids, 2 globes, and 24 Harderian glands) and 18 unilateral (2 eyelids, 8 globes, and 8 Harderian glands) cases. Ophthalmic lesions were observed in 69.6% (110/158), 92 bilateral (24 eyelids, 44 eyes, and 24 salt glands) and 18 unilateral cases.

All animals with fibropapillomas in the visual apparatus also had fibropapillomas in other parts of the body, such as fins, neck, plastron, and carapace. The macroscopic characteristics of these neoplasms ranged from nodules to sessile, pedunculated masses, sometimes with a verrucous appearance, and in some cases with the involvement of adjacent structures, such as the conjunctiva and eyelids.

Microscopic analysis of the neoplastic lesions showed proliferation of epithelial cells with papillomatous formation, and scarce fibrovascular or fibropapillomatous stroma, abundant fibrovascular stroma, with marked cellular hyperplasia, hyperkeratosis, acanthosis and, in some cases, intranuclear inclusion bodies that were observed in some epithelial cells, hydropic degeneration of the epidermis (external surface) and conjunctiva. In these lesions, there was a predominance of intratumoral spirorchid eggs, associated with predominantly lymphoplasmacytic inflammatory infiltrate in the stroma.

The most frequent lesion on the eyelids was fibropapilloma (20.2%; 32/158), followed by caseous discharge (7.6%; 12/158) and yellowish plaques or scar tissue on the skin and mucocutaneous junction (5%; 8/158).

**FIGURE 1:**
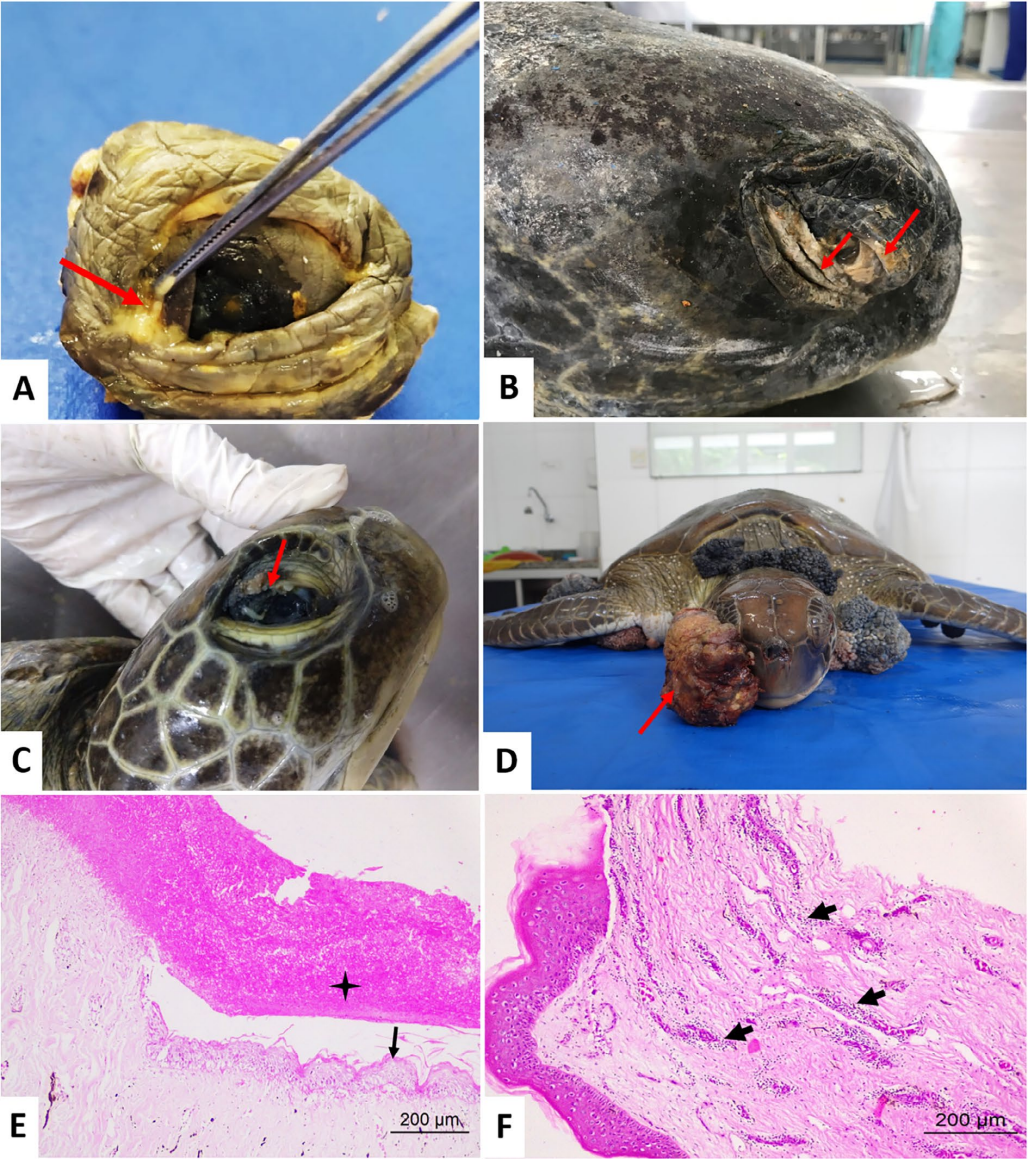
Lesions on the eyelids of *Chelonia mydas* . (A) Blepharitis. Note the caseous discharge. (arrow). (B) Scar tissue in the eyelid and conjunctiva of an adult *C. mydas* (arrows). (C, D) *Fibropapillomas on eyelids*. (C) Fibropapilloma in the upper eyelid (arrow). (D) Frontal view of the neoplasm with an irregular surface, exophytic growth, ulcerated (arrow). (E) Necrotic tissue (star) and hyperplasia of the conjunctival epithelium (arrow). (F) Photomicrograph of the eyelid of *Chelonia mydas* . *Viral conjunctivitis*. Perivasculitis. Moderate perivascular inflammatory infiltrate in the conjunctival stroma (arrows) and hyperplasia and hyperkeratosis of the epithelium (arrows). HE–Obj. 10×, 40×.

The exudative lesions revealed parasitic infection by spirorchid eggs at microscopy, with perivasculitis, hyperemia, migration of melanomacrophages, lymphocytes, eosinophils, and heterophils. In the scar tissue, acanthosis, areas of fibrosis, and mixed inflammation were observed. In the bulbar conjunctiva, fibropapillomatosis was also the predominant lesion (24%; 38/158), followed by cases of conjunctivitis (7.6%; 12/158). The changes observed in the eyelids and bulbar conjunctiva are illustrated in Figure 1.

The evaluation of eye samples identified fibropapillomatous lesions (14.5%; 23/158) located in the cornea and limbus. Phthisis bulbi (7.6%; 12/158), panophthalmitis (3.8%; 6/158), caseous secretion and scar tissue in the bulbar conjunctiva and sclera (both 2.5%; 4/158), and a perforated eye (2.5%; 4/158).

In the histopathology of eyes with fibropapillomas, neoplastic proliferation with migration of melanocytes in the region was observed. Intratumoral parasite eggs, keratitis, and parasitic uveitis were observed in 6.9% (11/158) of the evaluated samples. Anterior synechiae and secondary glaucoma were also identified as lesions.

**FIGURE 2:**
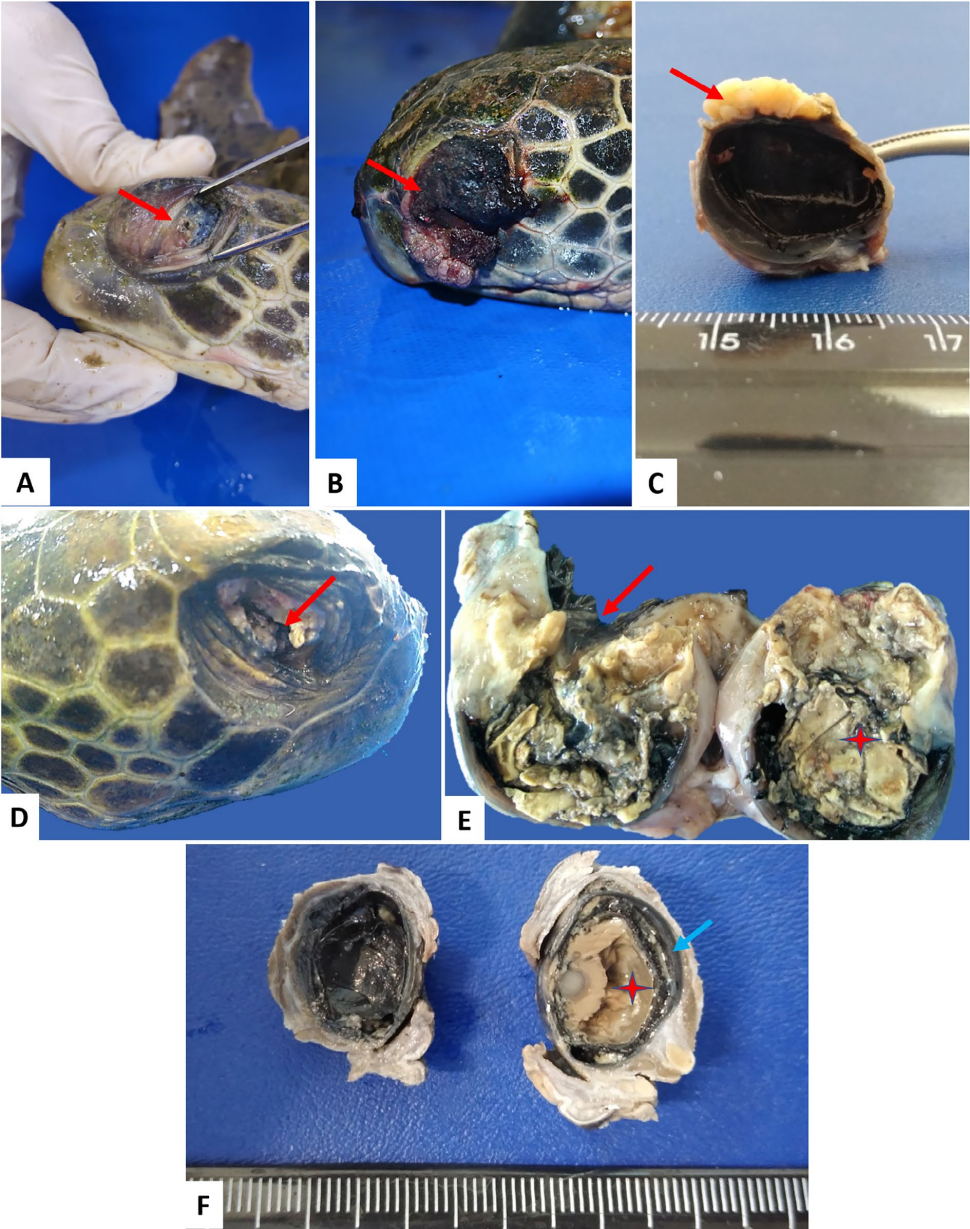
Morphological findings of ocular diseases in *Chelonia mydas* . (A) Anterior synechia and perforated eye (arrow). (B, C) Ocular fibropapilloma (arrow). (C) Longitudinal section of an eye with fibropapilloma attached to the cornea. (D, E) Necrotizing panophthalmitis. Perforated cornea (arrows), intraocular necrotic material (star). (F) Section in the sagittal plane of the eye with phthisis bulbi: Observe extensive caseous material in the anterior and posterior chambers with thickening of the uvea.

**FIGURE 3:**
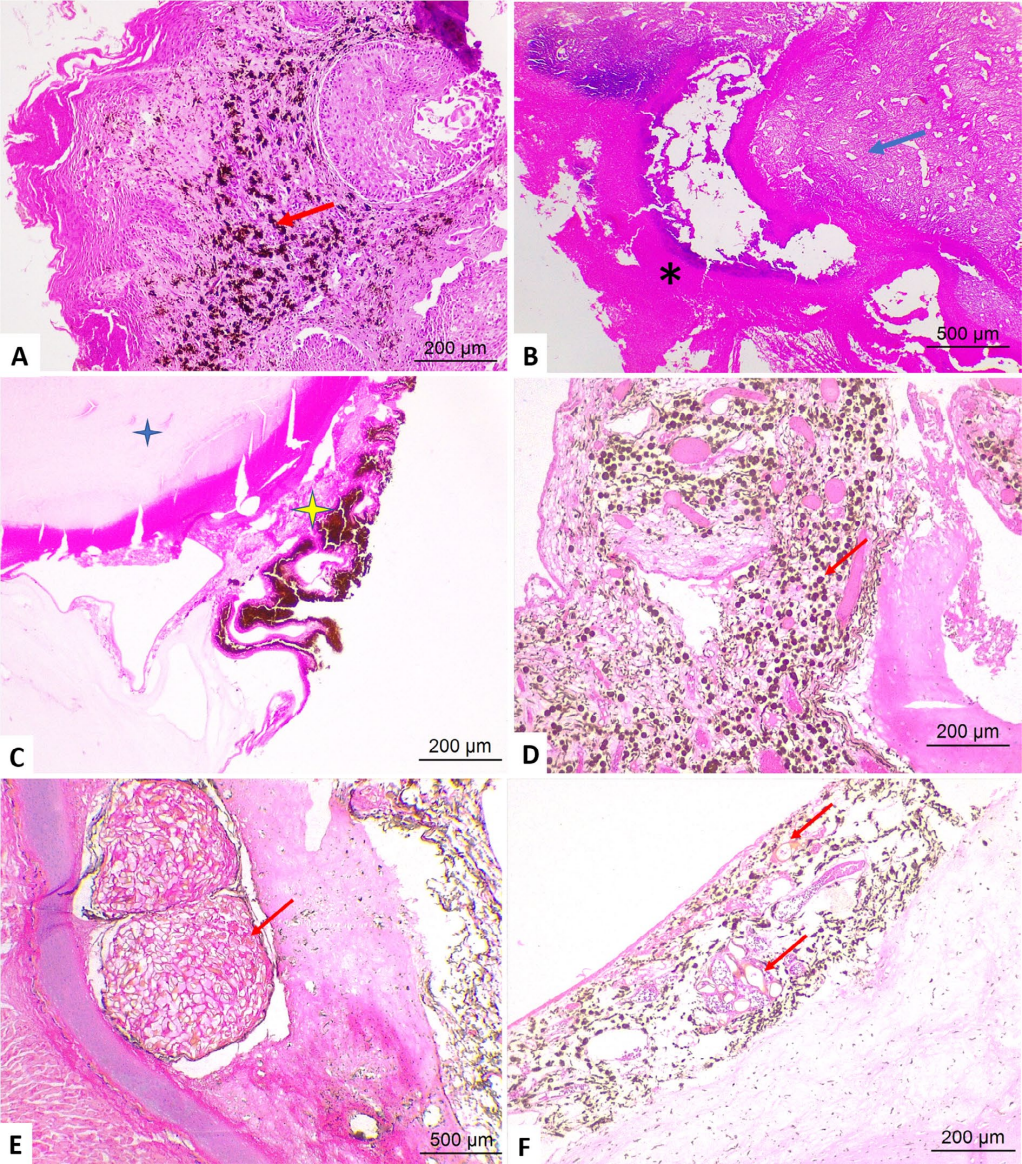
Photomicrographs of ophthalmic lesions in the eyes of *Chelonia mydas* . (A) Ocular fibropapillomatosis. Cornea—intratumoral melanocyte migration (arrow). (B) Necrotizing panophthalmitis. Necrotic tissue (asterisks) adhered to fibrovascular tissue (arrows). (C) Lens (blue star) and iris (yellow star) showing posterior synechia (arrow). (D) Migration of melanomacrophages (arrows) to the uveal tract. *Ocular spirorchidiasis—Parasitic granulomas*. (E) Sclera—granuloma with numerous spirorchid eggs (arrow). (F) Choroid parasitic granulomas, spirorchid eggs (arrow). HE staining–Obj. 4×, 10×.

Parasitic infection was also observed in samples from eyes without fibropapillomatosis, characterized by a parasitic granulomatous reaction due to the migration of spirorchid eggs into the cornea, scleral cartilage, and uvea (parasitic keratitis, scleritis, and uveitis). An adult parasite compatible with a spirorchid was present in the space between the uvea and sclera. Secondary glaucoma was one of the lesions identified, as were anterior synechiae (Figures 2 and 3).

In cases of phthisis bulbi, microscopy showed atrophy and disorganization of ocular structures, hyperplasia of the bulbar conjunctiva, keratitis, and hyperkeratosis. Eyes with panophthalmitis presented corneal ulceration, scleral hemorrhage, anterior and posterior synechia, hyperemia of the choroidal vessels; some cases had closure of the iridocorneal angle, characterizing a secondary glaucoma; and migration of inflammatory cells, melanomacrophages, melanocytes, lymphocytes, and heterophils (Figures 2 and 3).

Macroscopically, the salt glands showed on the cut surface, parasitic lesions characterized by multifocal blackened areas (19.6%; 31/158), purulent exudate, and calculi (both with 2.5%; 4/158). A juvenile female had dozens of white calculi of varying sizes located in the orbital cavity with an appearance similar to salt. Multifocal blackened areas were observed upon examination of this animal's Harderian gland on the surface and at the cut area, and millimetric calculi were possibly obstructing the glandular duct. This *C. mydas* had poor body condition, cachexia, hydroceloma, hydropericardium, and parasitic infection by spirorchids and specimens of *Neoctangium* sp. identified in the parasitological examination.

**FIGURE 4:**
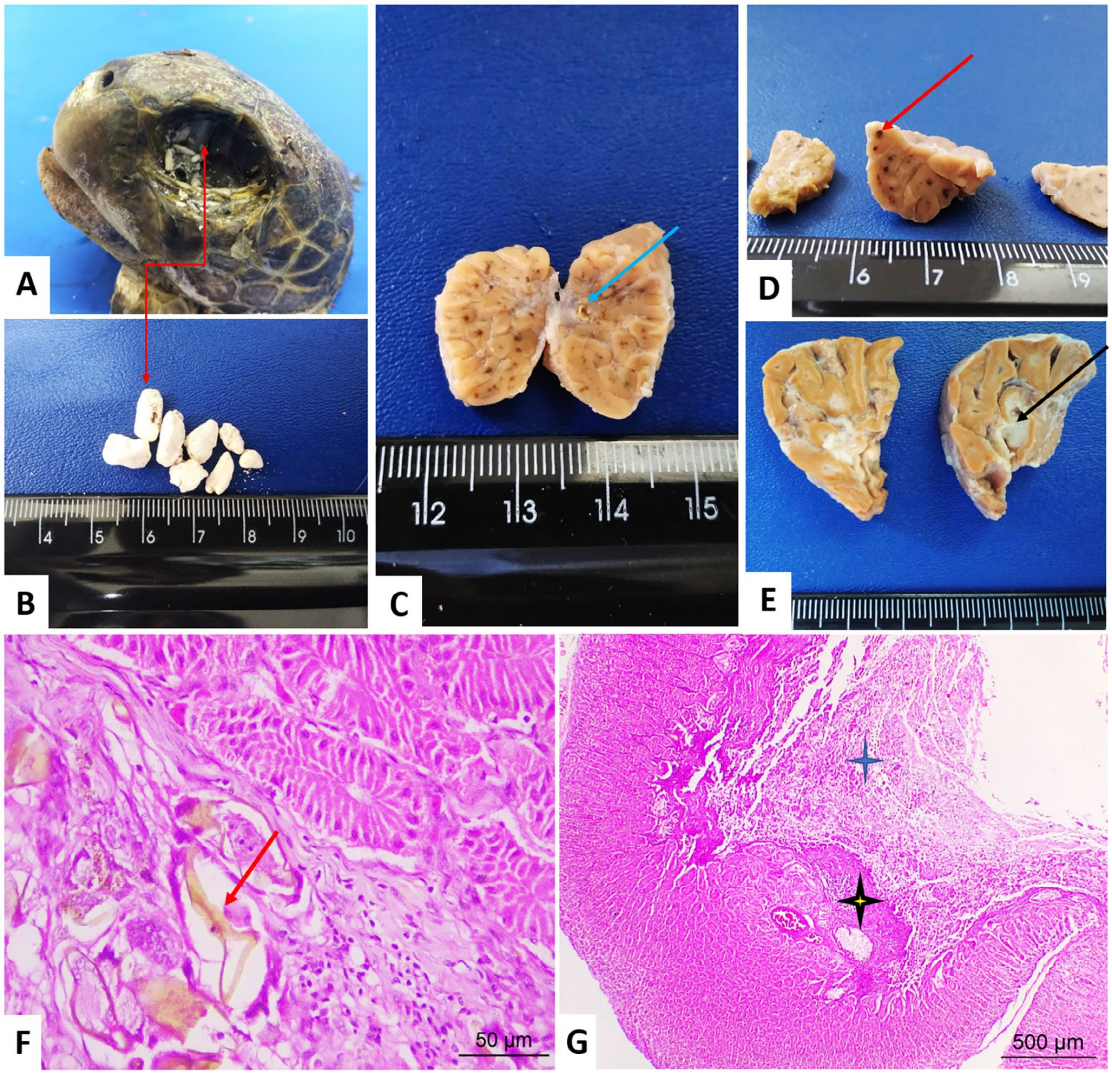
Lesions observed in the visual apparatus of *Chelonia mydas* . (A, B) Lithiasis. Several salt calculi in the orbital cavity (double red arrow). (C–E) Salt gland cut surface. (C) Intraparenchymal stone (blue arrow). (D) Multifocal blackened areas (red arrow). (E) Purulent exudate (black arrow). *Parasitic adenitis*. (F) Parasitic granuloma in the centrilobular region showing the spirorchid eggs (arrow). *Fibrinosuppurative adenitis*. (G) Centrilobular region with large amounts of inflammatory exudate (blue star) and areas of fibrosis (yellow star). HE staining–Obj. 4× and 40×.

The lesions located at microscopy in the centrilobular and periglandular region were configured as parasitic adenitis and/or periadenitis (spirorchid eggs). This condition was also observed in glands without macroscopic alteration (6.3%; 10/158). The inflammatory process was mixed, consisting of melanomacrophages, lymphocytes, heterophils, plasmocytes, and fibrinocaseous exudate. Two cases (1.3%) with blackened multifocal areas did not have spirorchids, characterized by a mild inflammatory infiltrate of heterophils associated with melanomacrophages. In the glands with calculi, there were areas of multifocal mineralization (Figure 4).

The spirorchid eggs observed in all parasitic lesions in the eyes and adnexa were predominantly type 1; in some cases, they were also associated with type 2 and 3 eggs.

Table 1 presents in detail the alterations according to the anatomical structures and the number of affected animals. A summary of the main changes is described in Table 2.

**TABLE 1:** Ophthalmopathies in *Chelonia mydas* stranded on the north coast of Bahia.

Anatomical structures	Region	Morphological diagnosis	Total	(%)	*N*°
Eyelids	Skin	Fibropapillomatosis	15	46.9	9
		Scar tissue	8	25	4
	Mucocutaneous junction	Fibropapillomatosis	9	28.1	5
	Conjunctiva	Fibropapillomatosis	8	25	4
		Mucopurulent conjunctivitis	12	37.5	6
		Parasitic conjunctivitis	8	25	4
Eyes	Cornea	Fibropapillomatosis	20	32.2	12
		Keratitis	8	13	4
		Ulcerative keratitis	2	3.2	2
	Limbus	Fibropapillomatosis	3	4.8	2
	Conjunctiva	Fibropapillomatosis	38	61.3	23
		Conjunctivitis	12	19.3	6
	Sclera	Parasitic scleritis	7	11.3	5
	Uvea	Parasitic choroiditis	16	25.8	10
		Previous synechia	3	4.8	3
		Closure of the iridocorneal angle (secondary glaucoma)	4	6.4	4
	All structures	Panophthalmitis	6	9.7	6
		Phthisis bulbi	12	19.3	12
Salt gland	Centrilobular	Parasitic adenitis	28	43.7	17
	Periglandular	Parasitic periadentitis	12	18.7	7
	Glandular duct	Lithiasis	4	6.2	3

*Note:* (%) percentage according to the *N* of each structure (32 eyelids, 62 eyes and 64 salt glands).

Abbreviation: *N*, number of animals.

**TABLE 2:** Summary of changes in the visual apparatus of *Chelonia mydas* .

	Total	Number of animals
Total study samples	158	39
Without changes	48	20
Fibropapillomatosis	93	24
Spirorchidiasis	74	31
Phthisis bulbi	12	12
Perforated eye	4	4
Lithiasis	4	3

## 4 Discussion

Studies exploring and describing ocular diseases in sea turtles are infrequent. In this context, to our knowledge, the study presented here is the first to characterize ocular diseases in green turtles in Brazil and the second to correlate spirorchidiasis with ophthalmic lesions.

The results of this study show fibropapillomatosis as the main lesion observed in the visual apparatus of *C. mydas* . It is a debilitating disease of a multifactorial nature, with the main etiological agent being *Chelonid Alphaherpesvirus 5* (ChHV5), characterized by the formation of single or multiple tumors, with a warty, smooth, or rough appearance, pigmented or not, in various regions of the body, especially in soft tissues [16, 17]. The presence of these tumors in this anatomical region was described in previous studies developed in other Brazilian states [18, 19].

Therefore, the high frequency of these neoformations in animals found in the northeast of Brazil is probably associated with several predisposing factors, such as viral and parasitic infections and exposure to pollutants in the marine environment, possibilities also considered in other regions of the country [19, 20], It is noteworthy that ChHV5 viral particles can be detected in blood, plasma, urine, cloacal, oral and ocular secretions [21, 22, 23]. In the region where the study was conducted, a case of viral infection was reported in a loggerhead turtle with bilateral mucoid secretion, chemosis, conjunctival hyperemia, and bilateral eyeball retraction [24]. The macro and microscopic characteristics observed in fibropapillomas corroborate the data described in the literature [9, 17, 25, 26]. All turtles affected by fibropapillomatosis were juveniles, confirming the study developed by Jones et al. [27].

Animals with ocular fibropapillomatosis may have reduced or total loss of vision, compromising their health conditions, making them vulnerable to the action of predators, and reducing their ability to feed [9, 28]. Despite the lack of data on the clinical history of these animals, particularly those with ophthalmic abnormalities, it is believed that there is a strong correlation between visual loss and the systemic condition. This is largely due to the presence of large neoplasms. A study on ophthalmic lesions in turtles stranded due to the cold in North Carolina, United States of America, revealed ocular and periocular lesions, such as bilateral superficial ulcerative keratitis, perforating lesions, one case of phthisis bulbi, proliferative tissue in adnexa, and synechiae [8]. Interestingly, similar lesions were observed in the present study; however, with a different etiology, associated with parasitic and neoplastic processes and consequently nutritional disorders, such as anorexia and malnutrition.

Other important ophthalmic lesions are represented by inflammatory processes caused by various pathogenic agents such as bacteria, viruses, parasites, and protozoa. Isler et al. [5] evaluated loggerhead turtles for diseases and diagnosed five ocular lesions: catarrhal conjunctivitis, caseous conjunctivitis, keratitis, blepharitis, and corneal perforation. All the mentioned lesions were observed in the present study with *C. mydas*.

In addition to the mixed inflammatory processes, trematode eggs were observed in different structures of the visual apparatus, such as the palpebral conjunctiva, cornea, sclera, and choroid. Recently Jerdy et al. [29] reported the presence of spirorchid eggs in the choroid and optic nerve in sea turtles of the species *C. mydas* , *C. caretta* , and *L. olivacea* , and concluded that animals with ocular lesions caused by spirorchids are approximately 300% more likely to be thin or cachectic than animals with this parasite, but without ocular damage. In *C. mydas* from the north coast of Bahia, 12.8% (5/39) had poor body condition or cachexia.

Regarding the location of the parasitic lesions, similar to other studies, the choroid was one of the most affected regions [29, 30]. However, other anatomical sites were also involved, such as the sclera and cornea, with a predominance of type 1 eggs. The presence of eggs in unreported sites emphasizes the ability of these eggs to migrate within the vascular system of these animals [30, 31, 32, 33, 34].

The observed cases of lithiasis, particularly in the one case that demonstrated dozens of calculi within the orbit and the salt gland, indicate a failure in the solute dissolution function, an alteration that can occur in severely dehydrated sea turtles [9, 35].

Salt glands are important as they assist in osmoregulation by excreting solutes through the tear ducts. Active transport via the sodium‐potassium pump draws salt from the blood into the glandular parenchyma. This process is necessary because reptilian kidneys are significantly less efficient in osmoregulation when compared to mammalian kidneys [3, 36, 37, 38]. These stones present as hard, rough deposits associated with necrosis of surrounding tissues [9].

According to Flint et al. [9] granulomatous adenitis due to spirorchid eggs, as observed in most of the salt glands of the animals in this study, presents variable severity, although these are lesions commonly observed in the stroma that surrounds the central channels of the lobules and that can cause obstruction if spread to other regions.

## 5 Conclusion

The anatomohistopathological evaluation of the specimens revealed predominantly neoplastic lesions, with fibropapillomatosis being the most frequent, followed by inflammatory lesions, caused especially by spirorchidiasis.

Studies related specifically to ophthalmic changes in *C. mydas* are infrequent, and it is necessary to develop research focused on this topic.

## Authors' Contributions

**Danielle Nascimento Silva:** conceptualization, formal analysis, investigation, methodology, project administration, validation, writing – original draft. **José Luís Catão‐Dias:** investigation, resources, writing – original draft, writing – review and editing. **Marcelo de Souza Perinotto:** investigation, resources, writing – original draft, writing – review and editing. **Matheus Vilardo Loés Moreira:** formal analysis, validation, writing – review and editing. **Nayone Lantyer‐Araujo:** formal analysis, writing – review and editing. **Pedro Enrique Navas Suárez:** formal analysis, writing – review and editing. **Gustavo Rodamilans Macedo** – formal analysis, visualization, writing – review and editing. **Thaís Pires:** formal analysis, visualization, writing – review and editing. **Arianne Pontes Oriá:** investigation, resources, writing – original draft, writing – review and editing. **Alessandra Estrela‐Lima:** conceptualization, investigation, project administration, supervision, validation, writing – original draft, writing – review and editing.

## Disclosure

The authors have not used AI to generate any part of the manuscript. *Bioethics and Biosecurity Committee Approval*: The research protocols were approved by the Animal Welfare and Ethics Committee of the School of Veterinary Medicine and Animal Science of the Federal University of Bahia (protocol n°90/2018), in accordance with the Ministry's Biodiversity Information Authorization System of the Environment of Brazil—SISBIO (process n°64518‐1 and n°64518‐3) and certified by the National System for the Management of Genetic Heritage and Associated Traditional Knowledge—SISGEN (registration n° A0F349F) (Annexes).

## Conflicts of Interest

The authors declare no conflicts of interest.

## Supporting information


**Table S1:** General sample data.

## Data Availability

The data that support the findings of this study are available from the corresponding author upon reasonable request.
