# Supplementary material for: Anatomopathological Characterization of the Main Ocular Lesions in Green Turtles ( Chelonia mydas ) Along the Northern Coast of Bahia, Brazil
Source: Vet Ophthalmol. 2025 Nov 10;29(2):e70106. doi: 10.1111/vop.70106 (PMC12968750; doi:10.1111/vop.70106)
Supplement: Supplementary file 1 — Table S1: General sample data. [file VOP-29-0-s001.doc]

**SUPPLEMENTARY TABLE 1** General sample data

| **Animals** | **Species** | **Curved carapace length (mm)** | **Curved carapace width (mm)** | **Weight (kg)** | **Chronological category** | **Sex** | **Analyzed samples** |
| --- | --- | --- | --- | --- | --- | --- | --- |
| **TC1** | *C. mydas* | 381.00 | 335.00 | 6.15 | Juvenile | Female | Salt gland (OD; OS) |
| **TC2** | *C. mydas* | 357.00 | 337.00 | 3.57 | Juvenile | Male | Eyes; Salt gland (OD; OS) |
| **TC3** | *C. mydas* | 366.00 | 340.00 | 4.50 | Juvenile | Male | Eyes; Eyelids; Salt gland (OD; OS) |
| **TC4** | *C. mydas* | 331.00 | 306.00 | 2.78 | Juvenile | Female | Eyes; Salt gland (OD; OS) |
| **TC5** | *C. mydas* | 350.00 | 334.00 | 2.73 | Juvenile | Female | Eyes; Eyelids; Salt gland (OD; OS) |
| **TC6** | *C. mydas* | 417.00 | 370.00 | 5.54 | Juvenile | Female | Eyes; Salt gland (OD; OS) |
| **TC7** | *C. mydas* | 545.00 | 510.00 | 15.4 | Juvenile | Male | Salt gland (OD; OS) |
| **TC8** | *C. mydas* | 400.00 | 357.00 | 6.25 | Juvenile | Female | Eyes; Salt gland (OD; OS) |
| **TC9** | *C. mydas* | 365.00 | 335.00 | 2.82 | Juvenile | Female | Eyes (OD; OS) |
| **TC10** | *C. mydas* | 356.00 | 315.00 | 3.56 | Juvenile | Female | Salt gland (OD; OS) |
| **TC11** | *C. mydas* | 364.00 | 323.00 | 5.00 | Juvenile | Male | Eyes; Eyelids; Salt gland (OD; OS) |
| **TC12** | *C. mydas* | 350.00 | 310.00 | 3.70 | Juvenile | Female | Eyes; Salt gland (OD; OS) |
| **TC13** | *C. mydas* | 370.00 | 350.00 | 3.55 | Juvenile | Female | Salt gland (OD; OS) |
| **TC14** | *C. mydas* | 370.00 | 350.00 | 3.55 | Juvenile | Female | Salt gland (OD; OS) |
| **TC15** | *C. mydas* | 346.00 | 300.00 | 3.83 | Juvenile | Male | Salt gland (OD; OS) |
| **TC16** | *C. mydas* | 620.00 | 560.00 | 20.0 | Juvenile | Female | Eyes; Eyelids; Salt gland (OD; OS) |
| **TC17** | *C. mydas* | 655.00 | 570.00 | 28.4 | Juvenile | Female | Eyes; Salt gland (OD; OS) |
| **TC18** | *C. mydas* | 665.00 | 640.00 | 23.4 | Juvenile | Female | Eyes; Eyelids; Salt gland (OD; OS) |
| **TC19** | *C. mydas* | 400.00 | 310.00 | 7.63 | Juvenile | Male | Eyes; Eyelids; Salt gland (OD; OS) |
| **TC20** | *C. mydas* | 376.00 | 334.00 | 3.79 | Juvenile | Female | Salt gland (OD; OS) |
| **TC21** | *C. mydas* | 1.107 | 1.085 | 178 | Adult | Female | Eyes; Eyelids; Salt gland (OD; OS) |
| **TC22** | *C. mydas* | 590.00 | 490.00 | 13.00 | Juvenile | Female | Eyes; Eyelids; Salt gland (OD; OS) |
| **TC23** | *C. mydas* | 385.00 | 340.00 | 4.13 | Juvenile | Female | Eyes; Salt gland (OD; OS) |
| **TC24** | *C. mydas* | 313.00 | 300.00 | 3.55 | Juvenile | Male | Eyes; Eyelids; Salt gland (OD; OS) |
| **TC25** | *C. mydas* | 500.00 | 450.00 | 9.85 | Juvenile | Female | Eyes; Eyelids; Salt gland (OD; OS) |
| **TC26** | *C. mydas* | 358.00 | 300.00 | 3.70 | Juvenile | Female | Eyes; Salt gland (OD; OS) |
| **TC27** | *C. mydas* | 440.00 | 415.00 | 7.20 | Juvenile | Male | Eyes; Salt gland (OD; OS) |
| **TC28** | *C. mydas* | 380.00 | 330.00 | 3.86 | Juvenile | Female | Salt gland (OD; OS) |
| **TC29** | *C. mydas* | 333.00 | 305.00 | 3.60 | Juvenile | Male | Eyes; Eyelids; Salt gland (OD; OS) |
| **TC30** | *C. mydas* | 385.00 | 345.00 | 4.78 | Juvenile | Female | Eyes (OD; OS) |
| **TC31** | *C. mydas* | 322.00 | 314.00 | 3.16 | Juvenile | Female | Eyes (OD; OS) |
| **TC32** | *C. mydas* | 420.00 | 389.00 | 5.90 | Juvenile | Female | Eyes; Eyelids (OD; OS) |
| **TC33** | *C. mydas* | 405.00 | 365.00 | 7.67 | Juvenile | Female | Eyes; Eyelids (OD; OS) |
| **TC34** | *C. mydas* | 370.00 | 307.00 | 3.96 | Juvenile | Female | Eyes (OD; OS) |
| **TC35** | *C. mydas* | 380.00 | 350.00 | 6.24 | Juvenile | Female | Eyes; Eyelids; Salt gland (OD; OS) |
| **TC36** | *C. mydas* | 440.00 | 378.00 | 6.32 | Juvenile | Male | Salt gland (OD; OS) |
| **TC37** | *C. mydas* | 450.00 | 410.00 | 10.60 | Juvenile | Female | Eyes; Salt gland (OD; OS) |
| **TC38** | *C. mydas* | 440.00 | 385.00 | 6.77 | Juvenile | Female | Eyes; Eyelids; Salt gland (OD; OS) |
| **TC39** | *C. mydas* | 1.218 | 1.124 | 182 | Juvenile | Female | Eyes; Eyelids; Salt gland (OD; OS) |
